# Manual Sampling and Video Observations: An Integrated Approach to Studying Flower-Visiting Arthropods in High-Mountain Environments

**DOI:** 10.3390/insects11120881

**Published:** 2020-12-11

**Authors:** Marco Bonelli, Andrea Melotto, Alessio Minici, Elena Eustacchio, Luca Gianfranceschi, Mauro Gobbi, Morena Casartelli, Marco Caccianiga

**Affiliations:** 1 Department of Biosciences, University of Milan, 20133 Milano, Italy; alessio.minici@studenti.unimi.it (A.M.); elena.eustacchio@unimi.it (E.E.); luca.gianfranceschi@unimi.it (L.G.); morena.casartelli@unimi.it (M.C.); marco.caccianiga@unimi.it (M.C.); 2 Department of Environmental Science and Policy, University of Milan, 20133 Milano, Italy; mel8@hotmail.it; 3 Centre of Excellence for Invasion Biology, Department of Botany and Zoology, Stellenbosch University, Stellenbosch 7600, South Africa; 4 Section of Invertebrate Zoology and Hydrobiology, MUSE–Science Museum, 38122 Trento, Italy; mauro.gobbi@muse.it; 5 BAT Center–Interuniversity Center for Studies on Bioinspired Agro-Environmental Technology, University of Naples ‘Federico II’, 80138 Napoli, Italy

**Keywords:** Alps, *Androsace brevis*, endemism, flowering plants, insect behavior, plant–arthropod interactions, mountain ecology, pollination biology, pollinators, video

## Abstract

**Simple Summary:**

Our study compared two different methods to identify the arthropods visiting the flowers of the vulnerable endemic alpine species *Androsace brevis* (Primulaceae) and investigate their behavior. Using the traditional method of manual sampling we could taxonomically identify visiting arthropods on a fine scale and determine which taxa carry pollen. Conversely, video observations provided information on arthropod behavior and activity. By integrating the results obtained from these two approaches, we estimated the diversity of *A. brevis* flower-visiting arthropods and evaluated which taxa could be involved in its pollination. Our results, in addition to providing new insights on flowering plant–arthropod interactions in early season in the Alps, might be useful in developing effective methods of studying the ecological relationships in high-mountain ecosystems.

**Abstract:**

Despite the rising interest in biotic interactions in mountain ecosystems, little is known about high-altitude flower-visiting arthropods. In particular, since the research in these environment can be limited or undermined by harsh conditions and logistical difficulties, it is mandatory to develop effective approaches that maximize possibilities to gather high-quality data. Here we compared two different methods, manual sampling and video observations, to investigate the interactions between the high-mountain arthropod community and flowers of *Androsace brevis* (Primulaceae), a vulnerable endemic alpine species with a short flowering period occurring in early season. We manually sampled flower-visiting arthropods according to the timed-observations method and recorded their activity on video. We assessed differences and effectiveness of the two approaches to estimate flower-visiting arthropod diversity and to identify potential taxa involved in *A. brevis* pollination. Both methods proved to be effective and comparable in describing the diversity of flower visitors at a high taxonomic level. However, with manual sampling we were able to obtain a fine taxonomic resolution for sampled arthropods and to evaluate which taxa actually carry *A. brevis* pollen, while video observations were less invasive and allowed us to assess arthropod behavior and to spot rare taxa. By combining the data obtained with these two approaches we could accurately identify flower-visiting arthropods, characterize their behavior, and hypothesize a role of Hymenoptera Apoidea and Diptera Brachycera in *A. brevis* pollination. Therefore, we propose integrating the two approaches as a powerful instrument to unravel interactions between flowering plants and associated fauna that can provide crucial information for the conservation of vulnerable environments such as high-mountain ecosystems.

## 1. Introduction

Plant–arthropod interactions are an essential component of terrestrial ecosystems, sustaining life on Earth and providing vital ecosystem services. About 88% of existing flowering species, including many crops, are pollinated by animals, mostly insects [1,2,3]. Conversely, many arthropods depend on flowers for their existence. Almost all bees (Hymenoptera: Apoidea: Anthophila) are completely dependent on flowering plants for their life cycle [4], and many other anthophilous arthropods—e.g., flies (Diptera), thrips (Thysanoptera), and spiders (Araneae)—exploit flowers for multiple purposes, such as foraging, hunting, sheltering, mating, and oviposition [5,6,7,8]. Moreover, plant–arthropod interactions can even shape ecosystems, having cascading effects on their components [9,10,11,12]. However, these interactions are exposed to both natural and anthropogenic-related threats [13,14]. In particular, an emerging concern is the ongoing change in climate, which can affect flowering plants, arthropods, and their relationships in several ways [15,16,17,18,19]. For instance, climate change can alter the frequency and the intensity of extreme meteorological and hydrological events, leading to habitat loss and fragmentation; moreover, variations in temperature can alter the timing of key stages both in plants (e.g., flowering) and arthropods (e.g., emergence period) or lead some species to migrate according to their ecological needs, while the climate niche for species that cannot migrate can shrink or disappear [14,20].

In mountain ecosystems, climate warming is expected to cause differential phenological and altitudinal shifts in plants and anthophilous species, leading to modifications of biotic interactions and potential mismatches between flowering and flower-visiting arthropods [21,22,23,24,25,26,27,28]. Knowledge of the actors involved is essential to assess the precise impact of climate change on flowering plant–arthropod interactions and to predict its effects on mountain ecosystems, as well as to hypothesize habitat management strategies or develop effective conservation plans. However, despite the rising interest in such interactions in mountain ecosystems [26,29,30], there is still a lack of knowledge about flower-visiting arthropods in these rich and fragile ecosystems [31]. For instance, apart from some early works [32,33], only a few recent studies have dealt with flower-visiting species in the Alps [31,34,35,36,37,38,39,40,41]. This knowledge gap might partly exist because mountain environments are often erroneously considered unpolluted and undisturbed and because of a perceived lack of short-term economic interest in this topic. Moreover, mountain environments are generally characterized by harsh and unpredictable conditions that can give rise to several logistical difficulties during field work, often limiting or undermining research. For instance, working under extreme conditions, such as microclimatic instability and remoteness, entails facing multiple constraints, such as restricted time-windows or limited opportunities for sampling that can severely affect the outcomes of data collection in the field. Thus, when investigating ecological relationships in high-mountain ecosystems, it is mandatory to employ effective approaches that maximize possibilities to gather high-quality data.

Flower-visiting arthropods have traditionally been monitored by direct observations and manual sampling, where timed observations have been proposed and used as a standard, repeatable, and effective method [38,42,43,44,45,46,47,48]. Nonetheless, an emerging technique is video observation, which has been employed with various goals and approaches to study the flower-visiting arthropod communities [39,49,50,51,52,53]. Although the arthropods collected by manual sampling can be taxonomically identified on a fine scale and in some cases may offer the possibility to perform investigations on the nature of their interactions with the focal plant (e.g., assessing the presence of pollen on sampled specimens), arthropod behavior and activity on plants cannot be observed or described in detail using this method. Conversely, fine-scale information can be obtained by monitoring arthropod behavior and activity with noninvasive and nondisruptive methods, such as recording observations by video. Both of these approaches can provide insight concerning pollination ecology but, to our knowledge, no study has compared their effectiveness in the context of high-mountain environments.

In this study we therefore compare two different methodological approaches (i.e., traditional manual sampling and video observations) to investigate interactions between the high-mountain arthropod community and the flowers of a plant species during the anthesis (i.e., blooming period). We employed *Androsace brevis* (Hegetschw.) Cesati (Primulaceae) as model species, an alpine plant with a very early and short flowering period. The flowering occurs immediately after snowmelt, typically lasting about two weeks for single plants and ranging from two to three weeks for the entire population. We made this choice because in this moment of the season—when few trophic resources are available for arthropods and few taxa are active and can pollinate flowers—the potential impact of mismatches between plants and arthropods could be particularly relevant [24].

The present study aims to assess differences and relative effectiveness of manual sampling and video observations in (i) estimating the diversity of *A. brevis* flower-visiting arthropods and (ii) inferring arthropod interactions with *A. brevis*, identifying potential taxa involved in its pollination by means of both palynological analyses of pollen found on sampled arthropods and video analysis of their behavior. Finally, (iii) we discuss costs and benefits of the two methodologies and highlight advantages of an integrated approach in the context of the high-mountain environment.

## 2. Materials and Methods

### 2.1. Study Species

*A. brevis* is a narrow endemic cushion plant that grows above 2000 m asl on rocky ridges and peaks in a restricted area in the Southern Alps of Northern Italy (Lombardy) and adjacent Switzerland. The extent of occurrence is estimated at 907 km^2^, the area of occupancy at 92 km^2^. Populations of this species are scattered and of limited size and, following IUCN criteria, its conservation status is Vulnerable (VU) [54].

**Figure 1:**
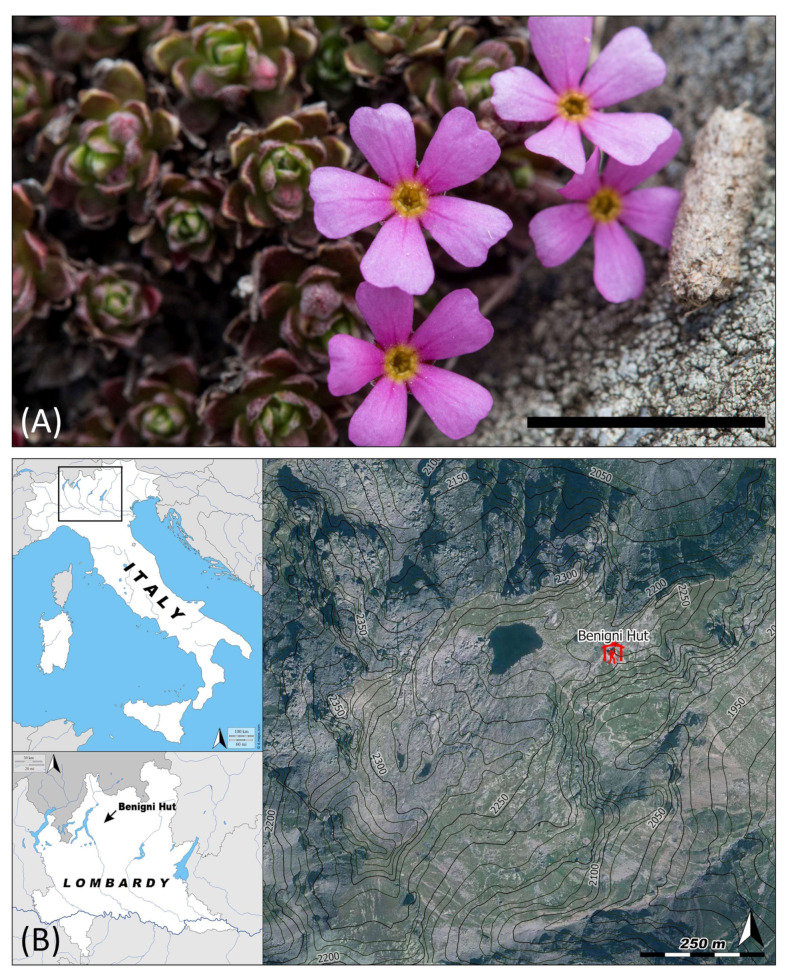
Study species (**A**) and site (**B**). *Androsace brevis* flowers, bar: 1 cm (**A**); and location of the Mountain Hut “Cesare Benigni” (**B**). Lombardy and Italy maps: www.d-maps.com, study area: Ortofoto AGEA 2012.

*A. brevis* usually flowers between the end of May and the beginning of June. Each cushion can produce from a few to about 200 solitary flowers held by 5- to 20-mm erect pedicels. The flower (Figure 1A) is about 4 mm long, characterized by a pink corolla (ca. 8 mm in diameter) with a yellow mouth (ca. 0.9 mm in diameter). The species is homostylous (i.e., flowers with styles of uniform length) and both stigma and anthers are located inside the corolla tube. The anthers are located slightly above the stigma, about 0.5 mm from the mouth of the corolla tube.

### 2.2. Study Site

The study was conducted in the Orobie Bergamasche Regional Park, a protected mountain area of the Orobic Alps (South-Eastern Alps) in the Alpine biogeographical region [55], in the vicinity of the Mountain Hut “Cesare Benigni” (Northern Italy, Lombardy, Province of Bergamo, UTM WGS84—32T E 543,496 N 5096577, 2222 m asl). The study area (Figure 1B) is characterized by the presence of a small glacial lake surrounded by rocky slopes with chasmophytic vegetation (EU Habitats Directive, Annex I habitat type, code 8220), including *A. brevis*. The population we studied occupies an area of approximatively 70,000 m^2^, which includes the plateau surrounding the hut, eastward to the lake, and nearby rocky outcrops and ridges, southward to the lake.

The climatic regime in the Orobic Alps is typically alpine, with oceanic climate influences promoting abundant rainfall distributed throughout the year. In particular, the mean annual temperature at the study site is 2.8 °C, while on average the precipitation regime amounts to 2190 mm per year (extrapolated from data of climatic stations: “Gerola Alta Pescegallo”, 1875 m asl, 1.5 km from the study site, observation period 1995–2018; “Mezzoldo Passo San Marco”, 1824 m asl, 5.5 km from the study site, observation period 2004–2018; and “Valtorta”, 982 m asl, 5 km from the study site, observation period 2012–2018).

The snow cover here usually lasts from October/November to May/June. Snow cover was monitored daily by checking the Mountain Hut webcam (available at https://orobiemeteo.com/) both to assess site accessibility and estimate the beginning of the *A. brevis* flowering season. The study was performed immediately after snowmelt, for three days between 8 and 16 June 2019, concurrently with the period of maximum flowering.

### 2.3. Manual Sampling

We manually sampled *A. brevis* flower-visiting arthropods according to the timed-observations method [42]. Each sampling session involved two researchers watching a single *A. brevis* cushion for one hour and collecting all flower-visiting arthropods (i.e., individuals touching at least one flower of the cushion). The researchers were crouched at opposite sides of the cushion, 50 cm apart, wearing grey clothes. This position both ensured a clear view of the flowers and allowed a prompt intervention, while minimizing disturbances for arthropods and avoiding restriction of their access to the flowers. We collected arthropods by mouth aspirator or, for flying insects, by placing a 50-mL tube with the opening downward over the individual and then tapping the tube with the insect inside. Equipment for net sampling was also available, but we did not have to use it since we were able to capture all the flower-visiting arthropods by using the aforementioned two methods. We placed each sampled arthropod in a 1.5-mL tube with 70% ethanol, immediately after the capture. As focal plants, we selected *A. brevis* cushions bearing at least five flowers at anthesis (mean ± standard error of the mean (SEM): 15.8 ± 4.3 flowers per cushion, ranging from 5 to 30 flowers). During each sampling day, two different pairs of researchers observed two cushions at the same time. We conducted sampling sessions between 10:00 and 18:00, but not under adverse circumstances, e.g., during rainfall (Table 1). Overall, during the three sampling days, we conducted 18 sampling sessions involving six different plants (Table 1).

### 2.4. Palynological Analyses

We performed quantitative palynological analyses of pollen grains found on sampled arthropods to assess which taxa can carry *A. brevis* pollen.

We vortexed each tube containing an ethanol-preserved specimen three times for 30 s to remove pollen grains from the arthropod body and to suspend pollen in the ethanol. Then, we removed the specimen from the tube and carefully observed it under a stereomicroscope to ensure that no pollen grains remained attached to the arthropod body. We repeated this process until no pollen grains were found on any specimen. Then, we placed the tubes containing the suspended pollen in a thermoblock at 65 °C to facilitate ethanol evaporation and concentrate pollen, while we shipped arthropods to expert taxonomists for morphological identification (Table S1).

We subjected the pollen samples to acetolysis [56], to make identification easier and more accurate. We poured 1 mL of the acetolysis mixture (acetic anhydride:sulfuric acid solution 9:1) into each tube containing pollen samples, we heated samples at 100 °C for 10 min and then placed in cold water to stop the acetolysis process. Subsequently, we centrifuged samples at 13,000× *g* for 20 min at 4 °C. We discarded supernatants and resuspended each pellet in distilled water. We repeated the last three steps (centrifugation, supernatant discarding, and pellet resuspension) until the supernatant was completely clean. Finally, after the last centrifugation cycle and removing the supernatant, we resuspended the precipitated pollen in 250 µL of a glycerol and distilled water mixture (1:1 v:v) and mounted it on microscope slides.

Moreover, during the field work, we collected in ethanol pollen samples from all plant species in flower within a radius of 500 m and within a difference in altitude of +/−200 m from the study site, in order to create a reference pollen library. We subjected these pollen samples to the same protocol described above, obtaining a library including 19 species (Table S2).

We observed pollen samples found on arthropods under an optical microscope (Leica DMRB) and identified pollen grains belonging to *A. brevis* with the support of the pollen reference library and pollen guides [57,58,59].

### 2.5. Video Observations

We video recorded the activity of flower-visiting arthropods on *A. brevis* cushions during the same three days of the manual sampling (Table 2). During video observations, we simultaneously recorded arthropod activity on three *A. brevis* cushions by means of three cameras (Olympus Tough TG-4, Olympus Tough TG-5, and Olympus OM-D E-M5 equipped with Olympus M.Zuiko Digital ED 12–50 mm f/3.5–6.3 EZ), ensuring the same video quality in macro-mode. Each video session lasted 1 h and included three 15-min videos per cushion. We selected this timing so as to have 5 min between each consecutive video to check camera function and change the batteries. We mounted each camera on a small tripod (h ≃ 40 cm) placed 40 cm from the focal cushion. At this distance we could obtain high-definition videos, while minimizing the disturbance for arthropods. During video recordings, researchers were 10 m away from the cameras and intervened only to check cameras or stop the ongoing observation and start a new one. As focal plants, we selected *A. brevis* cushions bearing at least five flowers at anthesis (mean ± SEM: 15.4 ± 3.1 flowers per cushion, ranging from 5 to 29 flowers). In total, we recorded 123 videos on nine different *A. brevis* plants.

### 2.6. Video Analysis and Behavioral Observations

We conducted video analysis using the software BORIS (version 7.7.3). BORIS is an open-source event-logging software, with which an ethogram of displays of interest can be built and the behavior of multiple subjects tracked on the basis of the codified displays [60]. The same researcher analyzed all the videos, observing each video multiple times to ensure replicability of display evaluation and to avoid missing observations. As for the manual sampling, for the video observations, too, we considered as flower-visiting arthropods the individuals touching at least one flower per cushion. Flower-visiting arthropod activity was monitored from the arrival on the first flower until the visitor left the field of view. As it was not possible to assign individual identification to visiting arthropods, we considered every individual entering the video as a new subject. Thus, during videos, we determined the number of observations of different subjects for each taxon, rather than the actual number of individuals, as the possibility that the same individual entered the video multiple times could not be excluded. The term “taxa” defined the lowest taxonomic levels that could be distinguished with certainty in all videos, and “undetermined” was associated with those arthropods that could not be identified with certainty (i.e., animals smaller than 1 mm, Figure S1). For most of the arthropods the lowest identifiable category was the order, while in some cases taxon was distinguishable at the suborder or superfamily level.

To infer the type of interaction existing among arthropods and *A. brevis*, we quantified the presence of flower-visiting arthropods (i.e., the number of subjects per taxon) and, for each subject, we recorded the occurrence of every entrance event inside the corolla tube of the flowers (hereafter “entrance”), a behavior that increases the probability of pollination. Only when arthropods entered the corolla tube or inserted their head or mouthparts for at least 1 s we did consider the behavior as “entrance”. On the basis of these data, we calculated two different dependent variables describing arthropod activity on an *A. brevis* cushion: the percentage of entering subjects among taxa during the video (calculated as the ratio of subjects performing at least one entrance over the total subjects observed), and the percentage of flowers entered per plant among taxa (the number of entered flowers by subjects over the number of flowers at anthesis on the cushion).

### 2.7. Data Analysis

**Figure 2:**
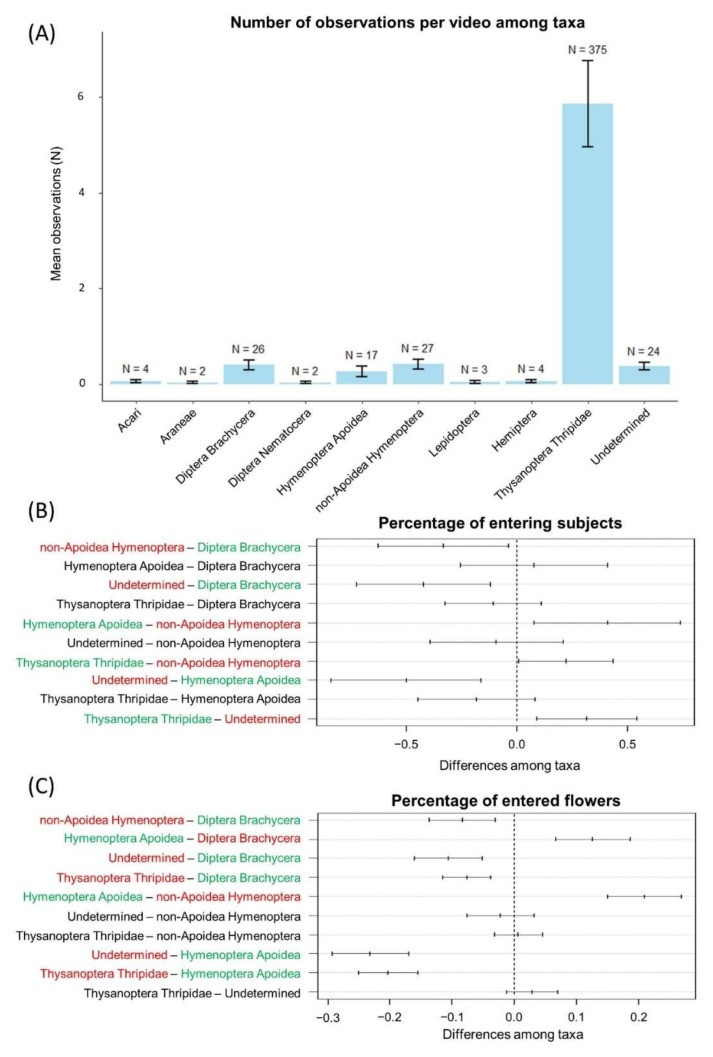
Results of video sampling (**A**) and behavioral analysis (**B**,**C**). Barplots (**A**) show the mean number of subjects observed per video for each taxon (the cumulative number of subjects observed is indicated above each bar). Error bars indicate standard error of the mean. Figure (**B**,**C**) show results of post hoc pairwise comparisons (Tukey’s test) for the percentage of entering subjects per video and the percentage of flowers entered per plant, respectively. Segments represent confidence intervals on the differences between the means for behavioral parameters of taxa couples. Segments crossing the central vertical line indicate nonsignificant differences, while noncrossing segments stands for significant ones. Left-shifted segments indicate reduction in the mean value of the behavioral parameter in the first element of taxa couple, whereas right-shifted segments represent an increase in the mean in the first taxon compared to the second one. Taxa pairs with no significant differences in the mean value of the behavioral parameter are reported in black, while for significant differences, green stands for taxa showing a relative increase in the behavioral parameter and red stands for those showing a decrease.

We assessed behavioral differences among arthropods using ANOVA. We built separate models using the percentage of entering subjects or the percentage of flowers entered as a dependent variable and taxa as a fixed factor. We excluded from the analyses the taxa with an insufficient number of observations (i.e., Acari, Araneae, Hemiptera, Diptera Nematocera, and Lepidoptera—Figure 2A, Table S3). We used Tukey’s post hoc test to perform pairwise comparisons among different taxa. Despite its likely taxonomic heterogeneity, the “undetermined” group was also considered in this analysis to assess whether taxa other than those identified are potentially involved in pollination.

To compare the number of taxa observed with the two methods (i.e., manual sampling and video observations), we built taxa accumulation curves for both approaches by plotting the cumulative number of sampled taxa (*y*-axis) against the time of sampling (*x*-axis) for manual sampling and observed taxa vs. monitoring time for video observations. We built these curves on the basis of taxa identified during videos, as they constituted the lowest taxonomic category common to both approaches. However, we excluded animals for which identification was unreliable (undetermined group) from the video curve (Figure S1). Conversely, for the sampling curve we included an additional taxon (Collembola), as it was uniquely found during manual sampling. Because during the first day of field work, the Olympus OM-D E-M5 failed to record some videos we excluded the observations made with that camera from this analysis to ensure comparability between the two approaches. Moreover, since on the first day of field work the manual sampling was suspended at 16:30, we did not consider the videos recorded after this time for the accumulation curve. As a result, we built the accumulation curves by merging two simultaneous sessions, both for manual sampling and video observations. Overall, for both approaches, the cumulative time effort was 9 h of sampling/observation on 6 different cushions.

The number of flowers at anthesis is known to influence plant attractiveness for pollinators [61,62,63,64]; therefore, the mean number of flowers between plants selected for manual sampling and video observations were compared. We did not find any significant difference in the number of flowers at anthesis between the cushions chosen for the two approaches, neither when considering all plants examined (unpaired *t*-test, *p* = 0.943) nor when comparing plants used for accumulation curves only (unpaired *t*-test, *p* = 0.887).

We conducted all statistical analyses in R environment (R version 3.6.0).

## 3. Results

By manual sampling, we collected a total of 12 flower-visiting arthropod individuals, capturing specimens in 39% of sessions, with a mean ± SEM of 0.67 ± 0.23 sampled specimens per session. All the arthropods were identified to family level, eight (67%) to genus level, and five (42%) to species level. The collected specimens belonged to 7 different orders (Araneae, Entomobryomorpha, Poduromorpha, Thysanoptera, Hemiptera, Hymenoptera, and Diptera) and 11 families (Table 3). All the sampled Diptera belonged to Brachycera (Drosophilidae, Sphaeroceridae, and Phoridae) and the sampled Hymenoptera belonged to Apoidea (Halictidae).

Although we observed pollen on all collected taxa, we detected *A. brevis* pollen only on Halictidae (Hymenoptera). The pollen of *A. brevis* could be clearly distinguished. It is radially symmetrical, isopolar, tricolporate, and prolate. The colpi are long and narrow with a distinct margin. The endoapertures are circular or slightly irregular. The ornamentation of the hexine is mainly micro-reticulated and the number of perforations at the apocolpium is reduced.

Regarding video observations, we observed flower-visiting arthropods in 64 videos (52% of the recorded videos). In total, we observed 484 subjects, belonging to at least 7 different orders (Figure 2A, Table S3): Thysanoptera, Hemiptera, Hymenoptera, Diptera, Lepidoptera, Araneae, and at least one order belonging to the subclass Acari. Moreover, according to video observations we could distinguish between different Diptera (suborder level: Brachycera vs. Nematocera) and Hymenoptera (superfamily Apoidea vs. other Hymenoptera). All non-Apoidea Hymenoptera observed were winged insects. In total 24 subjects (5% of the total observations) could not be identified with certainty and therefore we classified them as “undetermined” (Figure S1). Among the subjects observed, the vast majority was represented by Thripidae (78% of all observations, Figure 2A). However, their presence was highly variable among different cushions, with two of them hosting most of the subjects observed for this taxon (Table S3). Apart from Thripidae, the most frequent taxa were Hymenoptera (N = 44; 17 Apoidea and 27 other Hymenoptera) and Diptera (N = 28; 26 Brachycera and 2 Nematocera), accounting, respectively, for 9% and 6% of the subjects observed (Figure 2A). Lepidoptera, Hemiptera, Araneae, and Acari visited flowers in fewer than four cases (Figure 2A); therefore, these taxa, as well as Diptera Nematocera, were excluded from behavioral analyses. However, apart from Lepidoptera, we never observed these taxa entering *A. brevis* flowers (Tables S4 and S5).

Both percentage of entering subjects per video and percentage of flowers entered per plant varied significantly among taxa (ANOVA, respectively, F_4_ = 7.37, *p* value < 0.001; F_4_ = 41.75, *p* value < 0.001). In particular, post hoc pairwise comparisons revealed that the percentage of visiting subjects was higher in Hymenoptera Apoidea than in Thripidae and in both Hymenoptera Apoidea and Diptera Brachycera than in non-Apoidea Hymenoptera; moreover, when compared to the undetermined group, the percentage of visiting subjects was higher in all considered taxa, except for non-Apoidea Hymenoptera (Figure 2B, Table 4). We did not find any significant difference between other taxa from the paired comparisons considered (Figure 2B, Table 4).

The number of flowers entered per plant was significantly higher in Hymenoptera Apoidea or Diptera Brachycera than in Thripidae, non-Apoidea Hymenoptera, and the undetermined group (Figure 2C, Table 4). In contrast, we found no significant difference in the percentage of flowers entered per plant between Thripidae and the undetermined group, non-Apoidea and the undetermined group, or Thripidae and non-Apoidea (Figure 2C, Table 4).

**Figure 3:**
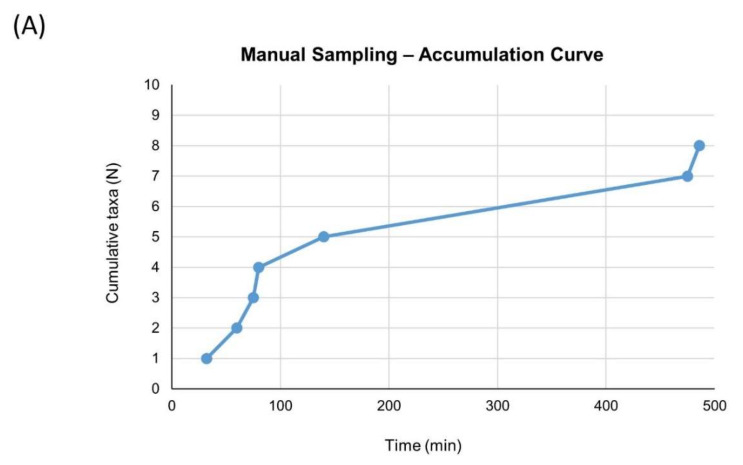

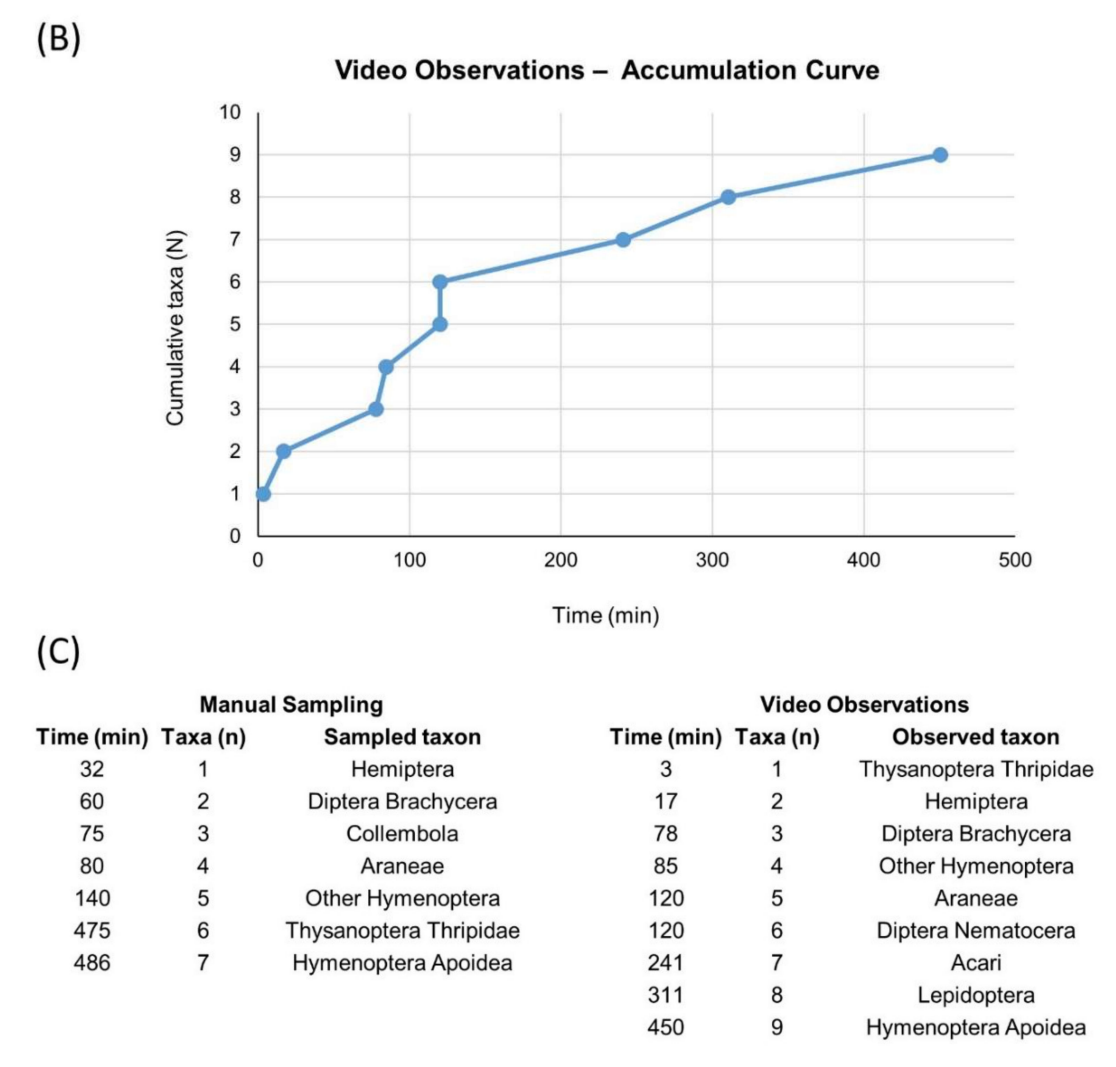
Accumulation curves for manual sampling (**A**) and video observations (**B**). Cumulative number of sampled (**A**) or observed (**B**) taxa on *y*-axis, time in minutes of sampling (**A**) or monitoring (**B**) on *x*-axis. Timing and identities of taxa are reported in (**C**).

The accumulation curves built for manual sampling (Figure 3A) and for video observations (Figure 3B) showed a similar trend, with a decrease in the rate of acquiring new taxa at about 100 min, and a maximum accumulation (7 for manual sampling and 9 for video observations) between 400 and 500 min.

## 4. Discussion

Our study offers new insights into the relationships between a vulnerable alpine flowering plant and the flower-visiting arthropod community and provides evidence of the importance of an integrated approach when investigating plant–arthropod interactions in the field, in particular in mountain environments.

First, we compared the information about the diversity of arthropods visiting *A. brevis* provided by two approaches, i.e., traditional manual sampling and video observations. Although there was a pronounced difference in the number of arthropods observed—possibly, at least in part, because of multiple observations of the same individuals during videos—the two approaches yielded accumulation curves with a similar trend, revealing a similar arthropod diversity at order level. Thus, the two methods proved effective and comparable in describing the flower-visitors’ diversity at a high taxonomic level. However, when looking at a finer scale, differences between the information provided by the two approaches were evident.

On the one hand, by manual sampling we could identify the flower-visiting arthropods at a taxonomic level that could not be achieved with video observations. For instance, we sampled a specimen belonging to Hymenoptera Apoidea, the best globally known and studied pollinators. It was possible to identify this individual as a Halictidae of the genus *Lasioglossum* Curtis, 1833, subgenus *Dialictus* Robertson, 1902. We were not able to determine the species of the specimen, however, because the species belonging to this subgenus are notoriously difficult to identify. Furthermore, the barcoding approach can hardly be applied: *Dialictus* is regarded as a “nightmare taxon” due to the absence of a well-defined barcode gap [66]. However, the level of information gained is far greater than that achievable with video observation, allowing us to identify the subgenus and sex of the specimen. Moreover, by manual sampling we were able to determine family and sometimes species of very small arthropods such as Collembola, which cannot be identified by video observations. The finer taxonomic resolution of sampled arthropods obtained with this approach made it possible to establish the basis for characterizing the flower-visiting community in depth, while the taxonomic level that could be achieved by video observation provides strongly simplified ecological information, not considering the large variety existing within the identified taxa.

On the other hand, it is important to note that the video observation approach may be less of a disturbance for visiting arthropods. Although we could not quantify visiting arthropods by employing this approach, the large number of subjects observed in videos (average of 15.74 per hour per plant) compared to individuals collected with manual sampling (average of 0.67 per hour per plant), and the simultaneous observations of multiple subjects during videos, never reached during manual samplings, indicate that video recording may have a minor impact on arthropod visitors compared to manual sampling. Moreover, with manual sampling we did not collect any Diptera Nematocera or Lepidoptera; however, these taxa were observed in videos, albeit with a low incidence. In conclusion, the video approach could be useful to spot even rare or elusive taxa. This could be particularly convenient in mountain environments, where there are only few sampling occasions and where, especially in early season, some taxa might be present but numerically not very abundant. Therefore, the integrated use of video observations and manual sampling can provide a complete description of the community of flower-visiting arthropods, avoiding loss of significant information.

Another important aspect that deserves to be highlighted is the evidence that the two approaches provided different and relevant insights about arthropods’ relationships with *A. brevis* flowers. In particular, we focused on identifying possible pollinators according to their behavior since pollination can be considered as one of the most critical biotic interactions in the alpine ecosystem, where most plants depend on insects for pollination, forming complex networks of dependencies among species [31]. One of the main advantages of manual sampling is the possibility to identify arthropod taxa carrying the pollen of the species of interest since, obviously, not all flower-visitors are pollinators. Nevertheless, many studies about pollination networks are based on observations of flower visits and few have tested whether the flower visitors actually carry pollen [67,68]. In the present study, we demonstrated that the pollen of *A. brevis* can be carried by Hymenoptera Apoidea. This result suggests that this taxon can play a role in the pollination of this species. However, due to the relatively small dimension of our sample, we cannot exclude that other taxa may be involved in the pollination. Conversely, video observation allowed us to characterize the behavioral ecology of the flower-visiting arthropods, monitoring their activity and time budget on flowers. Video analysis showed Hymenoptera Apoidea having a high percentage of entering subjects and the highest percentage of entered flowers, a further indication that these insects are good candidates in mediating *A. brevis* pollination. Similar to Hymenoptera Apoidea, Diptera Brachycera also showed high percentages of entering subjects and entered flowers. Indeed, these insects are alleged to play a major role as pollinators in the alpine environment [31,32,39], and according to their behavior we can hypothesize a potential role of Diptera Brachycera even in the absence of *A. brevis* pollen on the few individuals we sampled. Arthropods belonging to other taxa, such as arachnids and non-Apoidea Hymenoptera, entered flowers only occasionally, a behavior that, as expected for these taxa, makes them unlikely as pollinators. Thrips showed a relatively high percentage of entering subjects, similar to Brachycera dipterans and Apoidea hymenopterans, but with a significantly lower number of entered flowers. This behavior seems to suggest a certain fidelity to single flowers, which can potentially reflect territoriality, also supported by some apparently aggressive interactions between individuals of this taxon observed during video analyses. Territoriality and fighting were already observed in this taxon, presumably associated with mating, food, and guarding eggs [69], but this topic is still largely unexplored: video observations could be applied as a tool to shed light on these behaviors. Finally, some taxa were rarely observed approaching *A. brevis* flowers during videos and we could report their activity on this plant only anecdotally. Among them, lepidopterans always entered flowers multiple times, and thus they can potentially play a relevant role for *A. brevis* pollination. However, we cannot discriminate whether this limited occurrence is due to their behavior, mainly exploiting other flowering plants, for instance, or if it might depend on the low abundance in the study area and/or on a reduced overlap between *A. brevis* flowering and their activity.

In conclusion, although further research is needed to better elucidate the potential role and contribution of rarely sampled and observed taxa as pollinators of *A. brevis*; we could maximize data collection efficiency in a limited time frame by integrating the two approaches proposed in this study.

## 5. Conclusions

Despite their pros and cons, both investigated approaches, i.e., manual sampling and video observations, have proven to be suitable and useful for studying plant–arthropod interactions in a mountain environment, providing different but complementary data. Even in the restricted sampling period imposed by the very brief *A. brevis* flowering period and by mountain environmental conditions, we could both accurately identify arthropods visiting *A. brevis* flowers and also characterize their behavior by combining our data, revealing variability among taxa and allowing us to hypothesize their ecological roles. The integration of the two methods results in a synergistic approach that represents a powerful instrument to unravel relationships between flowering plants and associated fauna and can provide crucial knowledge for the conservation of vulnerable environments such as mountain ecosystems.

## Figures and Tables

**Table 1:** Time frame and effort of manual sampling. Day of sampling, time window, plant identity, the number of flowers at anthesis present on the cushion, and the number of samplings performed are reported.

	Manual Sampling			
Day	Time Window	Plant	N Flowers at Anthesis	N Sampling Sessions
8 June 2019	10:00–11:00	M1 M2	30 28	4 4
	12:00–13:00			
	14:00–15:00			
	15:30–16:30			
15 June 2019	15:00–16:00	M3 M4	5 10	3 3
	16:00–17:00			
	17:00–18:00			
16 June 2019	10:00–11:00	M5 M6	8 14	2 2
	12:00–13:00			

**Table 2:** Time frame and effort of video observations. Day of sampling, time window, plant identity, the number of flowers at anthesis present on the cushion, and the number of samplings performed are reported. The camera used for video recording is also indicated.

	Video Observations				
Day	Time Window	Plant	N Flowers at Anthesis	N Videos	Camera
		V1	14	23	Olympus Tough TG-5
8 June 2019	10:00–18:00	V2	24	23	Olympus Tough TG-4
		V3	29	20	Olympus OM-D E-M5
		V4	8	9	Olympus Tough TG-5
15 June 2019	15:00–18:00	V5	11	9	Olympus Tough TG-4
		V6	14	9	Olympus OM-D E-M5
		V7	28	10	Olympus Tough TG-5
16 June 2019	10:00–13:00	V8	5	10	Olympus Tough TG-4
		V9	6	10	Olympus OM-D E-M5

**Table 3:** Flower-visiting arthropods collected by manual sampling. Blank lines mean that no flower-visiting arthropods were collected. “na” means that taxonomic identification was not achieved. Nomenclature is according to Fauna Europaea [65]. * The *Lasioglossum* specimen is a female belonging to the subgenus *Dialictus.*

Day	Time Window	Plant	Class	Order	Family	Genus	Species
8 June 2019	10:00–11:00	M1					
		M2	Insecta	Hemiptera	Aphididae	*Cinara*	na
			Insecta	Diptera	Sphaeroceridae	*Leptocera*	*caenosa*
	12:00–13:00	M1	Entognatha	Entomobryomorpha (Collembola)	Entomobryidae	*Orchesella*	*arcuata*
			Insecta	Diptera	Drosophilidae	*Scaptomyza*	*pallida*
			Entognatha	Poduromorpha (Collembola)	Tullbergiidae	na	na
		M2	Insecta	Diptera	Phoridae	*Megaselia*	na
			Arachnida	Araneae	Linyphiidae	na	na
	14:00–15:00	M1	Insecta	Hymenoptera	Eulophidae	na	na
			Insecta	Hemiptera	Aphididae	*Rhopalosiphum*	*maidis*
		M2					
	15:30–16:30	M1	Entognatha	Poduromorpha (Collembola)	Onychiuridae	na	na
		M2					
15 June 2019	15:00–16:00	M3					
		M4					
	16:00–17:00	M3					
		M4					
	17:00–18:00	M3					
		M4					
16 June 2019	10:00–11:00	M5	Insecta	Thysanoptera	Thripidae	*Thrips*	*vulgatissimus*
		M6					
	12:00–13:00	M5					
		M6	Insecta	Hymenoptera	Halictidae	*Lasioglossum* *	na

**Table 4:** Difference in behavioral parameters among taxa visiting *A. brevis* flowers. Results of post hoc pairwise comparison (Tukey’s test) for all possible taxa pairs are reported. Taxa with an insufficient number of video observations were not included in these analyses (see Materials and Methods). For both the percentage of entering subjects per video and percentage of flowers entered per plant, the significance value and difference in mean values for all pairwise comparison are reported. Significant differences are in bold.

Taxa	Percentage of Entering Subjects		Percentage of Flowers Entered	
	*p* Value	Difference	*p* Value	Difference
Other Hymenoptera—Diptera Brachycera	**0.018**	**−0.33**	**<0.001**	**−0.08**
Hymenoptera Apoidea—Diptera Brachycera	0.970	0.08	**<0.001**	**0.13**
Undetermined—Diptera Brachycera	**0.001**	**−0.42**	**<0.001**	**−0.11**
Thripidae—Diptera Brachycera	0.658	−0.11	**<0.001**	**−0.08**
Hymenoptera Apoidea—Other Hymenoptera	**0.007**	**0.41**	**<0.001**	**0.21**
Undetermined—Other Hymenoptera	0.916	−0.09	0.781	−0.02
Thripidae—Other Hymenoptera	**0.034**	**0.22**	0.992	0.01
Undetermined—Hymenoptera Apoidea	**0.001**	**−0.50**	**<0.001**	**−0.23**
Thripidae—Hymenoptera Apoidea	0.318	−0.18	**<0.001**	**−0.20**
Thripidae—Undetermined	**0.001**	**0.32**	0.290	0.03

## Supplementary Materials

The following are available online at https://www.mdpi.com/2075-4450/11/12/881/s1, Table S1: Taxonomists who identified the sampled arthropods, Table S2: List of plant species in flower present within a radius of 500 m and within a difference in altitude of +/−200 m from the study site, Table S3: Distribution among taxa of the total number of observed subjects on each A. brevis plant during video observations, Table S4: Variation in behavioral parameters among taxa visiting A. brevis flowers, Table S5: Variation in flower entrance among taxa visiting A. brevis flowers, Figure S1: Undetermined arthropods, whose taxon was not clearly identifiable from video recordings.
