# Supplementary material for: Manual Sampling and Video Observations: An Integrated Approach to Studying Flower-Visiting Arthropods in High-Mountain Environments
Source: Insects. 2020 Dec 11;11(12):881. doi: 10.3390/insects11120881 (PMC7764373; doi:10.3390/insects11120881)
Supplement: Supplementary file 1 [file insects-11-00881-s001.pdf]

Table S1. Taxonomists who identified the sampled arthropods.

**Taxon**	**Taxonomist (affiliation)**
Araneae	Paolo Pantini (Museo Civico di Scienze Naturali Enrico Caffi di Bergamo – Sezione di Zoologia degli Invertebrati, Italy)
Collembola	Barbara Valle (Università degli Studi di Milano – Dipartimento di Bioscienze, Italy)
Thysanoptera	Barbara Conti (Università di Pisa – Dipartimento di Scienze Agrarie, Alimentari e Agro-ambientali, Italy)
Hemiptera	Alice Casiraghi (Centro Mixto Universidad de Valencia – Instituto de Biología Integrativa de Sistemas, Spain; Universitat de Barcelona – Departament de Biologia Evolutiva, Ecologia i Ciències Ambientals, Spain),<br>Nicolas Pérez Hidalgo (Centro Mixto Universidad de Valencia – Instituto de Biología Integrativa de Sistemas, Spain; Museo de Ciencias Naturales de Barcelona – Departamento de Artrópodos, Spain)
Hymenoptera	Fabrizio Rigato (Museo Civico di Storia Naturale di Milano – Sezione di Entomologia, Italy)
Hymenoptera, Apoidea	Andree Cappellari (Università degli Studi di Padova – Dipartimento di Agronomia, Animali, Alimenti, Risorse naturali e Ambiente, Italy)
Diptera	Daniele Avesani (Museo di Storia Naturale di Verona – Sezione di Zoologia, Italy)
Diptera, Drosophilidae	Gerhard Bächli (Universität Zürich – Institut für Evolutionsbiologie und Umweltwissenschaften, Switzerland)
Diptera, Sphaeroceridae	Jindřich Roháček (Muzeum Śląskie – Entomologické oddělení, Czech Republic)
Diptera, Phoridae	Henry Disney (University of Cambridge – Department of Zoology, United Kingdom)

Table S2. List of plant species in flower present within a radius of 500 m and within a difference in altitude of +/-200 m from the study site. These species were sampled to create a reference pollen library.

**Species**	**Family**
Androsace brevis	Primulaceae
Androsace vandellii	Primulaceae
Anthyllis alpicola	Fabaceae
Cardamine hirsuta	Brassicaceae
Carex curvula	Cyperaceae
Daphne striata	Thymelaeaceae
Draba aizoides	Brassicaceae
Erica carnea	Ericaceae
Geum montanum	Rosaceae
Lotus corniculatus	Fabaceae
Myosotis alpestris	Boraginaceae
Polygala chamaebuxus	Polygalaceae
Polygala vulgaris	Polygalaceae
Primula hirsuta	Primulaceae
Pulsatilla alpina	Ranuncolaceae
Ranunculus sp.	Ranuncolaceae
Soldanella alpina	Primulaceae
Viola biflora	Violaceae
Viola sp.	Violaceae

Table S3. Distribution among taxa of the total number of observed subjects on each *A. brevis* plant during video observations. The number of videos with flower-visiting arthropods per plant is also showed.

Plant	Day	N videos with active arthropods	Total observations							
			Diptera	Hymenoptera	Lepidoptera	Thripidae	Hemiptera	Araneae	Acari	Undetermined
V1	8 June 2019	19	6	8	0	66	1	1	1	2
V2	8 June 2019	23	19	16	2	305	2	0	0	12
V3	8 June 2019	2	1	0	1	0	0	0	0	0
V4	15 June 2019	3	1	0	0	0	0	0	3	2
V5	15 June 2019	5	1	3	0	0	1	0	0	2
V6	15 June 2019	1	0	0	0	0	0	0	0	1
V7	16 June 2019	4	0	10	0	2	0	1	0	0
V8	16 June 2019	5	0	5	0	2	0	0	0	5
V9	16 June 2019	2	0	2	0	0	0	0	0	0
TOTAL		64	28	44	3	375	4	2	4	24

Table S4. Variation in behavioral parameters among taxa visiting *A. brevis* flowers. The total number of subjects observed together with mean value and standard error of the mean calculated for the percentage of entering subjects per video and for the percentage of flowers entered per plant are reported.

Taxa	Total observed subjects	Percentage of entering subjects		Percentage of flowers entered	
		mean	SEM	mean	SEM
Diptera Brachycera	26	0.92	0.05	0.14	0.02
Diptera Nematocera	2	0	0	0	0
Hymenoptera Apoidea	17	1.00	0	0.26	0.06
Other Hymenoptera	27	0.59	0.10	0.05	0.01
Lepidoptera	3	1.00	0	0.24	0.15
Thripidae	375	0.82	0.02	0.06	0.00
Hemiptera	4	0	0	0	0
Araneae	2	0	0	0	0
Acari	4	0	0	0	0
Undetermined	24	0.50	0.10	0.03	0.01

Table S5. Variation in flower entrance among taxa visiting *A. brevis* flowers. For all taxa the total number of subjects observed is reported along with the total number of flowers entered during all observations and the number of entrances per subject (mean ± SEM).

Taxa	Total observed subjects	Number of flowers entered		
		**total**	**mean**	**SEM**
Diptera Brachycera	26	70	2.69	0.34
Diptera Nematocera	2	0	0	0
Hymenoptera Apoidea	17	53	3.12	0.76
Other Hymenoptera	27	31	1.15	0.26
Lepidoptera	3	18	6.00	3.51
Thripidae	375	496	1.32	0.06
Hemiptera	4	0	0	0
Araneae	2	0	0	0
Acari	4	0	0	0
Undetermined	24	12	0.50	0.10

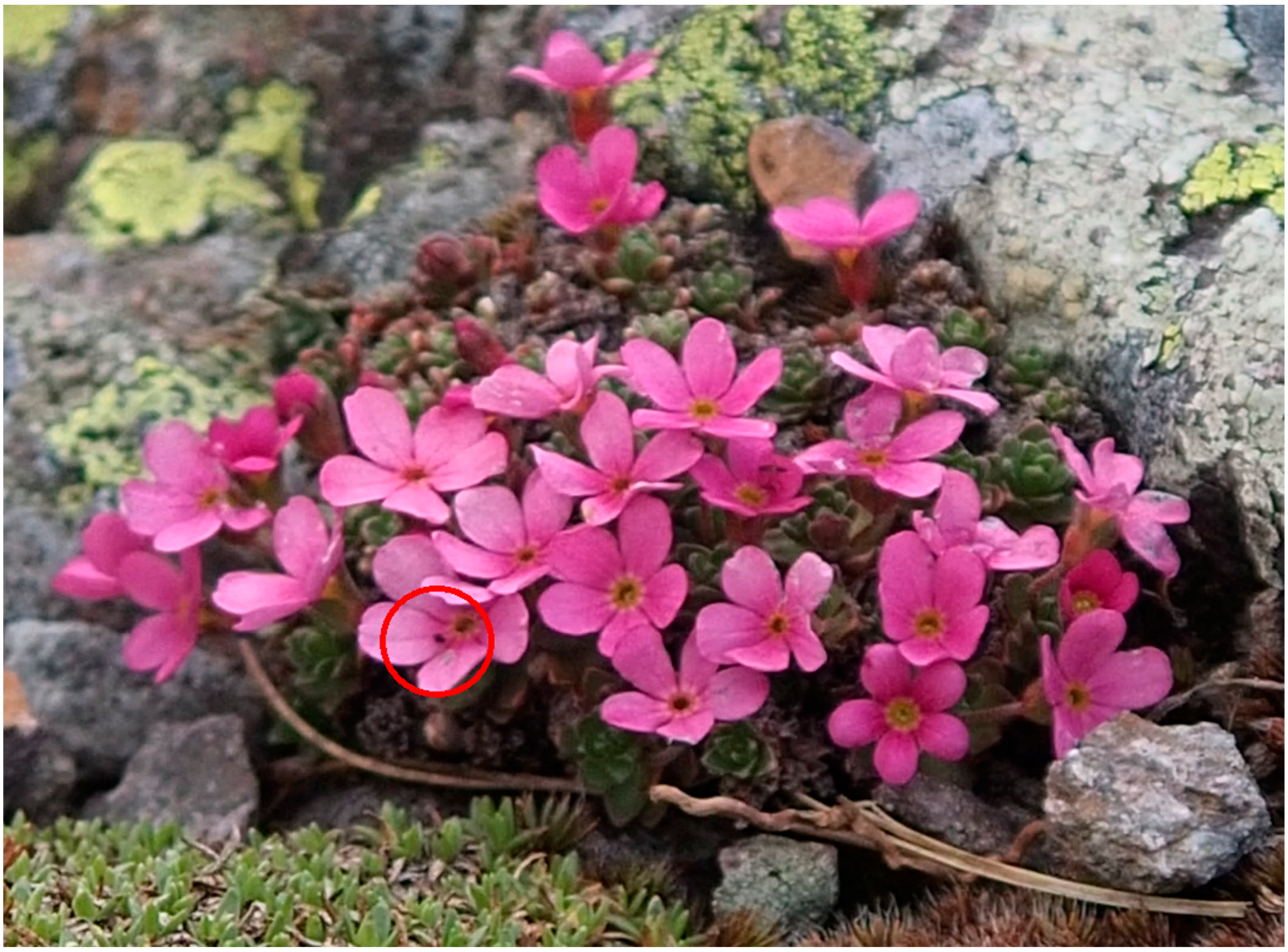

Figure S1. Undetermined arthropods, whose taxon was not clearly identifiable from video recordings. The figure represents the maximum-sized individual (inside the red circle) among the observed undetermined arthropods. These arthropods were very small (less than 1mm) and often only detectable when moving (23 out 24 subjects).
